# Plasmonic Bound States in the Continuum to Tailor Exciton Emission of MoTe_2_

**DOI:** 10.3390/nano13131987

**Published:** 2023-06-30

**Authors:** Yuxuan Jin, Kai Wu, Bining Sheng, Wentao Ma, Zefeng Chen, Xiaofeng Li

**Affiliations:** 1 School of Optoelectronic Science and Engineering & Collaborative Innovation Center of Suzhou Nano Science and Technology, Soochow University, Suzhou 215006, China; yxjin1106@stu.suda.edu.cn (Y.J.); 20214039004@stu.suda.edu.cn (K.W.); 20215239070@stu.suda.edu.cn (B.S.); 20214239012@stu.suda.edu.cn (W.M.); 2 Key Lab of Advanced Optical Manufacturing Technologies of Jiangsu Province & Key Lab of Modern Optical Technologies of Education Ministry of China, Soochow University, Suzhou 215006, China

**Keywords:** BIC, exciton, van der Waals materials, MoTe_2_

## Abstract

Plasmon resonances can greatly enhance light–matter interactions of two-dimensional van der Waals materials. However, the quality factor of plasmonic resonances is limited. Here, we demonstrate a plasmonic quasi-bound state in the continuum (quasi-BIC), which is composed of gold nanorod pairs. Through controlling the rotation angle of the nanorods, the quality factor of the plasmonic BIC mode can be tuned. Simulation results show that the plasmonic BIC combines the advantages of high-quality factor from the BIC effect and small mode volume from plasmonic resonance. Experiment results show that the designed plasmonic BIC mode exhibits a quality factor higher than 15 at the wavelength of around 1250 nm. Through integrating the plasmonic bound state structure with monolayer molybdenum ditelluride (MoTe_2_), the exciton emission of MoTe_2_ in the PL spectrum split into two exciton-polariton modes, which is attributed to the high Q factor and strong interaction between the BIC mode and excitons of MoTe_2_.

## 1. Introduction

Plasmonic resonances have attracted great attention due to their high confinement of light and enhancement of light–matter interactions. Localized plasmon resonances and propagating surface plasmon have been widely applied in photovoltaics [1,2], bio-sensors [3,4], photocatalysis [5,6], photodetectors [7,8,9,10] and so on. Recently, plasmonic resonances were also used for coupling with excitons of van der Waals semiconductors (e.g., MoS_2_, WSe_2_) to explore exciton–polariton strong coupling and excitonic devices (e.g., excitonic lasers) [11,12,13,14,15,16]. However, because of the heat loss of metals, the quality factor (Q factor) of plasmon resonances is always smaller than 10 in the visible and near-infrared light region [17] and the line width is larger than that of the excitons [18,19]. As a result, photoluminescence spectral broadening is mainly observed in the plasmon exciton coupling system and it is hard to directly observe mode splitting in spectrum [20,21,22].

Recently, the concept of the bound state in the continuums (BICs) was introduced from condensed state physics to nanophotonics. In a photonic system, BICs can support an infinite Q factor, which means the system cannot couple with the external electromagnetic wave [23,24,25,26,27]. After introducing a small perturbation to the symmetry of the BIC structure, the BIC mode can be tuned to a quasi-BIC mode. In this case, the electromagnetic wave outside the system can directly interact with the BIC structure, resulting in a finite Q factor [28,29,30,31,32,33,34]. So far, a quasi-BIC structure with an ultra-high Q factor has been designed based on low-loss dielectric material (e.g., silicon) for nanophotonic systems.

Here, we propose a quasi-bound state in the continuum in near-infrared regions based on the plasmonic resonances of gold nanorod pairs. Through controlling the rotation angle of the nanorods, the symmetry breaking can be manipulated so that the quality factor of the plasmonic BIC mode can be tuned. Different from the dielectric BIC structures, the electrical field is highly confined surrounding the surface of the metal nanorods, which provides a smaller mode volume. Experimentally, we achieved a plasmonic BIC mode at the wavelength of around 1250 nm with a Q factor higher than 15. When this plasmonic BIC structure was integrated with monolayer MoTe_2_, the plasmonic BIC mode could adequately interact with the excitons of MoTe_2_. Due to the high Q factor and strong interaction between the BIC mode and excitons, the exciton emission of MoTe_2_ was split into two modes in the photoluminescence spectrum.

## 2. Methods

Device fabrication: The gold nanorod array was fabricated on Si/SiO_2_ wafer using standard electron beam lithography and metal deposition process, followed by a PMMA film spin coating on the gold nanorod array as a supporting layer. Then, the gold nanorod array could be transferred onto a prepared quartz (or other substrate) using the wet transfer process.

Measurements: The infrared light was generated from the supercontinuum laser (NKT Photonics), then modulated with an acousto-optic tunable filter to achieved monochromatic light. The light spot was focused on the sample and an optical power meter was used to measure the intensity of transmission signal. The sample was measured with a micro-PL spectrometer with PMT detectors.

## 3. Result and Discussion

### 3.1. Simulation

**Figure 1:**
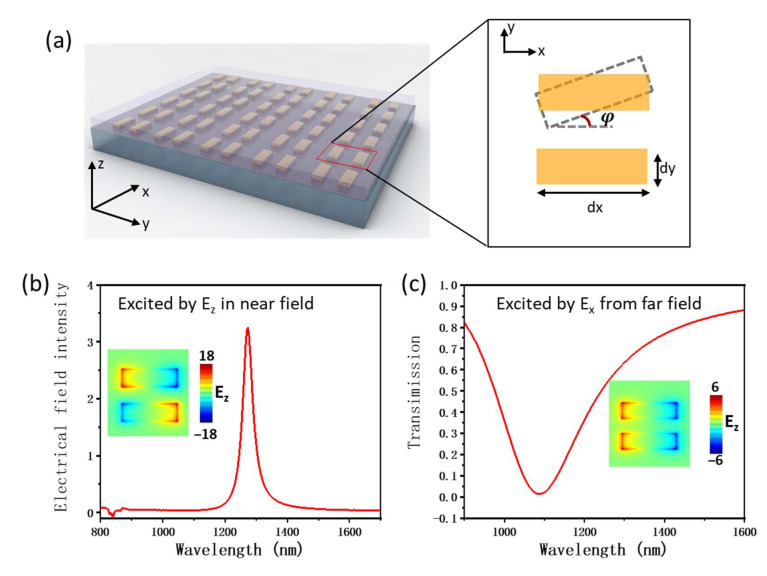
(**a**) Schematic figure of the array of gold nanorod pairs supporting the plasmonic BIC mode. The parameters of the gold nanorod pair are shown on the right, where d_y_ = 80 nm, d_x_ = 210 nm and *φ* is the rotation angle. The periodic parameters of the nanorod pair array are P_x_ = 400 nm and P_y_ = 600 nm. (**b**) The electrical field intensity ${\left|E\right|}^{2}$ of a probe placed 2 nm from surface one of the gold nanorods; meanwhile, a dipole source for excitation is placed on the terminal of the other gold nanorod. The inset shows the simulated Re(E_z_) patterns at the wavelength of 1280 nm. (**c**) The transmission spectrum of gold nanorod pairs being excited by plane wave incidence condition with Ex polarization. The inset shows the simulated Re(Ez) patterns at the wavelength of 1090 nm.

The structure of the plasmonic BIC is composed of a periodic array of gold nanorod pairs on quartz and covered with PMMA, as shown in Figure 1a. When the two gold nanorods are parallel, this structure can support symmetric and antisymmetric modes at different wavelengths. The antisymmetric mode exhibits opposite induced-currents in the two rods, which results in no net electrical dipoles coupling with the external electromagnetic wave (EMW). This mode can be considered as a BIC mode and cannot be excited by far field EMW. To observe the response spectrum of this BIC mode, a dipole source with Ez-polarization was set on the end facet of one nanorod to excite the antisymmetric resonance, and a probe was placed near the surface of one of the nanorods. Here, the numerical simulation was based on the commercial finite difference time domain (FDTD) software package (Ansys Lumerical 2020 R2.4). As shown in Figure 1b, we saw a narrow dip with a linewidth of 12 nm at the wavelength of 1280 nm, which corresponded with the Q factor of 100 (Q factor $\approx \frac{\lambda }{\Delta \lambda },$ where λ and Δλ are the center wavelength and the linewidth of the dip, respectively). The distribution of the electrical field (inset of Figure 1b) shows that the two nanorods exhibited the same electrical dipole resonance but with opposite phases. Therefore, the radiation in the far field was destructive interference and could not be detected. More importantly, the electrical field was highly confined at the surface of gold nanorods, which means a smaller mode volume can be achieved. In other words, the plasmonic BIC combines the advantages of high Q from the BIC effect and small mode volume from plasmonic resonance. For comparison, we used Ex-polarization light to excite the plasmons of nanorods from far field; the response spectrum is shown in Figure 1c. The resonance wavelength was around 1090 nm with a linewidth of about 100 nm. Obviously, the linewidth of the plasmonic BIC mode was about ten times smaller than that of the plasmonic resonance mode.

**Figure 2:**
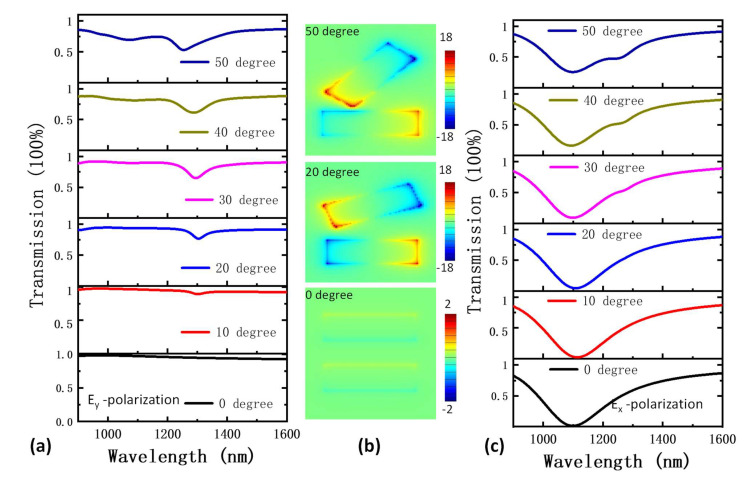
(**a**) Transmission spectra of plasmonic BIC structures being excited by plane wave incidence conditions with Ey polarization. (**b**) simulated Re(Ez) patterns of plasmonic BIC mode with nanorod rotation angles of 20 and 50 degrees at the dip wavelength in figure (**a**). For the rotation of 0 degrees, the simulation wavelength was set at 1280 nm. (**c**) Transmission spectra of plasmonic BIC structures being excited by plane wave incidence condition with Ex polarization.

Through introducing a small perturbation to the symmetry of the gold nanorod pairs, the BIC mode can be tuned into so-called quasi-BIC mode, which can directly interact with far field EMW [25,26,27]. Here, the perturbation of symmetry is controlled by rotating one gold nanorod of the pair by an angle *φ*. Figure 2a shows the transmission spectra of E_y_-polarization light with different rotation angles of gold nanorods. When the rotation angle was increased to 10 degrees, a narrow dip appeared at the wavelength of around 1280 nm, which was very close to the BIC mode. As the rotation angle increased, the linewidth of the dip was broadened slightly, while the depth became deeper. Ultimately, the dip was shifted to 1250 nm at the angle of 50 degrees. The electrical field distributions for the rotation angle of 0, 20 and 50 degrees are shown in (Figure 2b). For the 0 degree rotation angle, there was no electrical field enhancement found at the interface of the nanorods at the wavelength of around 1280 nm, because the BIC mode cannot be excited from far field. When this rotation angle was 20 degrees and 50 degrees, both the nanorod pairs exhibited electrical dipole resonance with opposite phases, like the BIC mode. However, as one of the nanorods was rotated, the condition for destructive interference in far field was no longer strictly satisfied. Therefore, this mode can be excited from far field. As the rotation angle was increased to 50 degrees, there was another broader dip at the wavelength of 1060 nm, which can be attributed to the plasmonic resonance mode of the rotated nanorod excited by the E_y_-polarization light. The transmission spectrum for E_x_-polarization light is shown in Figure 2c. The plasmonic resonance mode was clearly shown at the wavelength of 1080 nm with a linewidth of 210 nm. The simulated Re(E_z_) patterns of the plasmonic mode (Figure S1) for different rotations were almost the same with that for zero degrees, which is typical for electrical dipoles mode. When the rotation angle increased to 40 degrees, a slight dip was found at the wavelength of about 1260 nm, which can be attributed to the quasi-BIC mode.

### 3.2. Experiment Results

**Figure 3:**
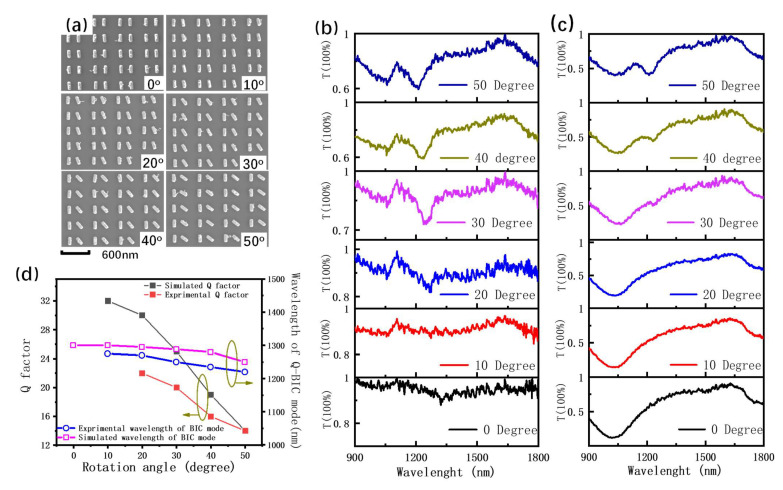
(**a**) SEM images of the gold nanorod pair arrays with different rotation angles. (**b**) Transmission spectra of the gold nanorod pair arrays under the incident light of E_y_-polarization. (**c**) Transmission spectra of the gold nanorod pair arrays under the incident light of E_x_ polarization. (**d**) Experimental and simulation Q factor and the wavelength of the plasmonic quasi-BIC mode extracted from the transmission dips.

Figure 3 shows the experiment results according to the above design parameters. The experimental transmission spectra agreed well with the simulation results. For E_y_-polarization light, the plasmonic quasi-BIC mode appeared when its rotation angle was higher than 20 degrees. As the rotation angle increased, the characteristic wavelength of the plasmonic quasi-BIC mode was little blue shifted from 1240 nm to 1210 nm and the Q factor of the quasi-BIC mode decreased from around 20 to 16, as shown in Figure 3d. At the large rotation angle (e.g., 50 degrees or 40 degrees), the Q factor was consistent with the simulation results. However, at small rotation angles, the experimental Q factor was lower than the simulation ones. One of the reasons was that the non-ideal edges (e.g., roughness) of the fabricated gold nanorods introduced additional loss for the system. For E_x_-polarization light, only plasmonic resonance mode was observed in the transmission spectra at small rotation angles, as shown in Figure 3c. The Q factor of the plasmonic resonance mode was about five, which was about three times lower than that of the plasmonic BIC mode.

### 3.3. Coupling with MoTe_2_

**Figure 4:**
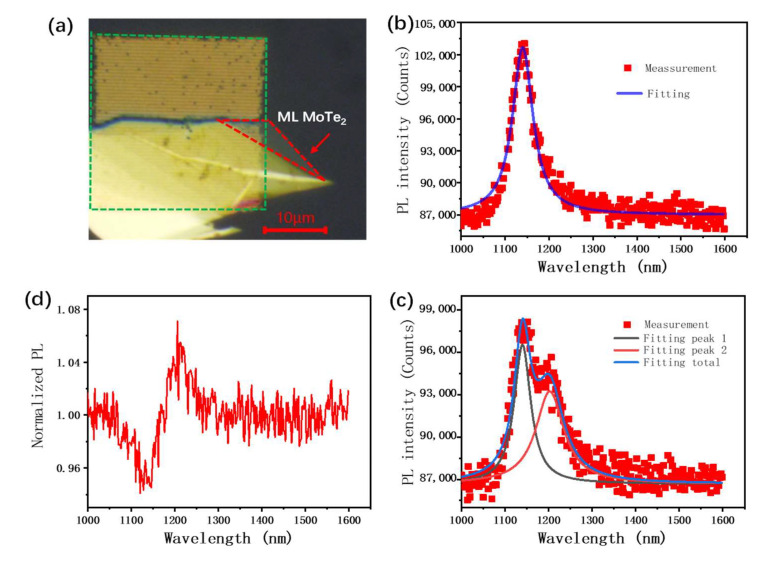
(**a**) Image of MoTe_2_ integrated with plasmonic quasi-BIC structure, in which the rotation angle of nanorods is 50 degrees. (**b**) PL spectrum data and the corresponding fitting curve of pure MoTe_2_. (**c**) PL spectrum data and the corresponding fitting curve of MoTe_2_ with plasmonic quasi-BIC structure. (**d**) The enhancement of PL intensity of MoTe_2_ integrated with plasmonic quasi-BIC structure.

Next, we adapted this quasi-BIC mode to couple with the exciton emission of monolayer MoTe_2_. The monolayer MoTe_2_ was mechanically exfoliated from bulk crystal on quartz and the array of gold nanorod pairs with a rotation angle of 50 degrees was transferred to the top of MoTe_2_. An image of MoTe_2_ integrated with the quasi-BIC structure is shown in Figure 4a. For comparison, we divided the monolayer MoTe_2_ into two parts: with and without the quasi-BIC structure. The whole structure was covered with PMMA film to protect the monolayer MoTe_2_ from the oxidation by air. The photoluminescence spectra of MoTe_2_ with and without the quasi-BIC structure are shown in Figure 4b,c. For pure MoTe_2_, there was one emission peak at the wavelength of 1144 nm, which corresponded to the exciton emission of monolayer MoTe_2_ [35,36]. For MoTe_2_ with the quasi-BIC structure, there were two peaks in the PL spectrum. The peak at the wavelength of 1205 nm corresponded to the plasmonic quasi-BIC mode, which had lower energy than the excitons of MoTe_2_. The other one, at the wavelength of 1141 nm, was very close to the exciton of MoTe_2_, but the intensity was lower than that of pure MoTe_2_. The PL mapping and the transmission spectrum of the device are shown in Figures S2 and S3. Figure 4d shows the enhancement of the PL spectrum (by dividing the PL spectrum of MoTe_2_ with and without the plasmonic structure). The enhancement of the PL spectrum was a Fano-like curve, which means that the plasmonic BIC mode not only directly enhanced the PL emission at the wavelength of the plasmonic mode, but also repartitioned the radiation channel of excitons through the exciton–plasmon coupling. The coupling system corresponded to the open cavity−exciton system, in which both excitons and plasmons can directly interact with the external field. It was different from excitons coupled with a closed cavity, such as the Fabry−Perot cavity, in which excitons are encapsulated within the cavity and cannot directly interact with the external field. Based on the cavity-quantum electrodynamic method, the total optical intensity of the hybrid exciton–plasmon system is given by: $I=I_{0}FM$, where $I_{0}$ is a typical Lorentz term describing the optical response of excitons uncoupled with plasmons, M is a Rabi term describing the Rabi splitting when the excitons are coupled with plasmons and F is a Fano function $F=\frac{{\left(\omega_{0}-\omega +q\right)}^{2}+{\gamma_{0}}^{2}}{{\left(\omega_{0}-\omega \right)}^{2}+{\gamma_{0}}^{2}}$. In the Fano function, $q=g/(\mu_{p}/\mu_{e})$, where *g* is the coupling strength between excitons and plasmons, $\frac{\mu_{p}}{\mu_{e}}$ presents the ration of coupling strength of the plasmons and excitons to the external field and $\gamma_{0}$ is the linewidth of the cavity mode. In the coupling system, the original exciton mode split into two exciton-polariton modes, which is described by Rabi term *M*. The low energy mode was dominated by plasmons, while the high energy mode was dominated by excitons. Then, the Fano term further redistributed the radiation channel through enhancing the low energy mode and reducing the high energy mode. In detail, after excitons excited in MoTe_2_, part of exciton energy transferred to the low energy mode and was emitted through the plasmon BIC mode, resulting in the lower emission intensity of the high energy mode. More discussion of this phenomenon is shown in Figure S4.

From the Fano function, it can also be seen that the energy redistribution depended on the coupling strength *g* and the linewidth $\gamma_{0}$ of plasmons. Higher *g* or lower $\gamma_{0}$ induced more energy transferring to the low energy mode. In our system, quasi-plasmonic BIC exhibited a higher Q (lower linewidth $\gamma_{0}$) than plasmonic mode. In addition, different from the dielectric BIC structure, the electric field of the plasmonic BIC mode was highly confined at the surface of gold nanorods, which resulted in an adequate interaction with excitons of MoTe_2_ and a higher coupling strength *g*. Therefore, the clear mode splitting in the PL spectrum and Fano line shape in the normalized PL spectrum were observed.

## 4. Summary

In summary, we designed a gold nanorod pair array supporting the plasmonic quasi-BIC mode in the near-infrared region. Simulation results show that the system can be tuned from BIC to quasi-BIC mode with a Q factor up to 100 by breaking the symmetry of the metal nanorods. This plasmonic quasi-BIC mode also shows high light-confinement at the surface of gold nanorods. Experiment results show that the Q factor of the plasmonic BIC mode is over 15, which is about four times higher than that of the plasmonic mode. Due to the high Q factor and strong interaction between the BIC mode and excitons of MoTe_2_, the exciton emission of MoTe_2_ in the PL spectrum split into two exciton-polariton modes, coupling monolayer MoTe_2_ with the plasmonic quasi-bound state. We believe that the plasmonic quasi-BIC with a high Q factor and small mode volume would provide promising nanophotonic structures to study strong coupling of exciton polaritons in two-dimensional van der Waals systems.

## Data Availability

All data supporting the findings of this study are available within the article and its Supplementary Information. All other data are available from the corresponding author upon reasonable request.

## Supplementary Materials

The following supporting information can be downloaded at: https://www.mdpi.com/article/10.3390/nano13131987/s1, Figure S1: The simulated Re (Ez) patterns of the plasmonic mode, Figure S2: PL mapping at the wavelength of MoTe_2_ exciton, Figure S3: the transmission spectrum of the ML MoTe^2^ covering by plasmonic BIC structure, Figure S4: The diagram for PL spectrum of MoTe^2^ without and with plasmonic BIC mode.
