# Supplementary material for: Plasmonic Bound States in the Continuum to Tailor Exciton Emission of MoTe2
Source: Nanomaterials (Basel). 2023 Jun 30;13(13):1987. doi: 10.3390/nano13131987 (PMC10343364; doi:10.3390/nano13131987)
Supplement: Supplementary file 1 [file nanomaterials-13-01987-s001.zip › nanomaterials-2425768-supplementary.pdf]

Supporting information for

# "Plasmonic Bound States in the Continuum to Tailor Exciton Emission of $MoTe_2$"

**Yuxuan Jin [1,2,†], Kai Wu [1,2,†], Bining Sheng [1,2], Wentao Ma [1,2], Zefeng Chen [1,2,*] and Xiaofeng Li [1,2,*]**

1 School of Optoelectronic Science and Engineering & Collaborative Innovation Center of Suzhou Nano
Science and Technology, Soochow University, Suzhou 215006, China; yxjin1106@stu.suda.edu.cn (Y.J.); 20214039004@stu.suda.edu.cn (K.W.); 20215239070@stu.suda.edu.cn (B.S.); 20214239012@stu.suda.edu.cn (W.M.)

2 Key Lab of Advanced Optical Manufacturing Technologies of Jiangsu Province & Key Lab of Modern
Optical Technologies of Education Ministry of China, Soochow University, Suzhou 215006, China

* Correspondence: zfchen86@suda.edu.cn (Z.C.); xfli@suda.edu.cn (X.L.);

† These authors contributed equally to this work.

Figure S1: The simulated Re(Ez) patterns of the plasmonic mode.

Figure S2: PL mapping at the wavelength of $MoTe_2$ exciton.

Figure S3: the transmission spectrum of the ML MoTe2 covering by plasmonic BIC structure.

Figure S4: The diagram for PL spectrum of MoTe2 without and with plasmonic BIC mode.

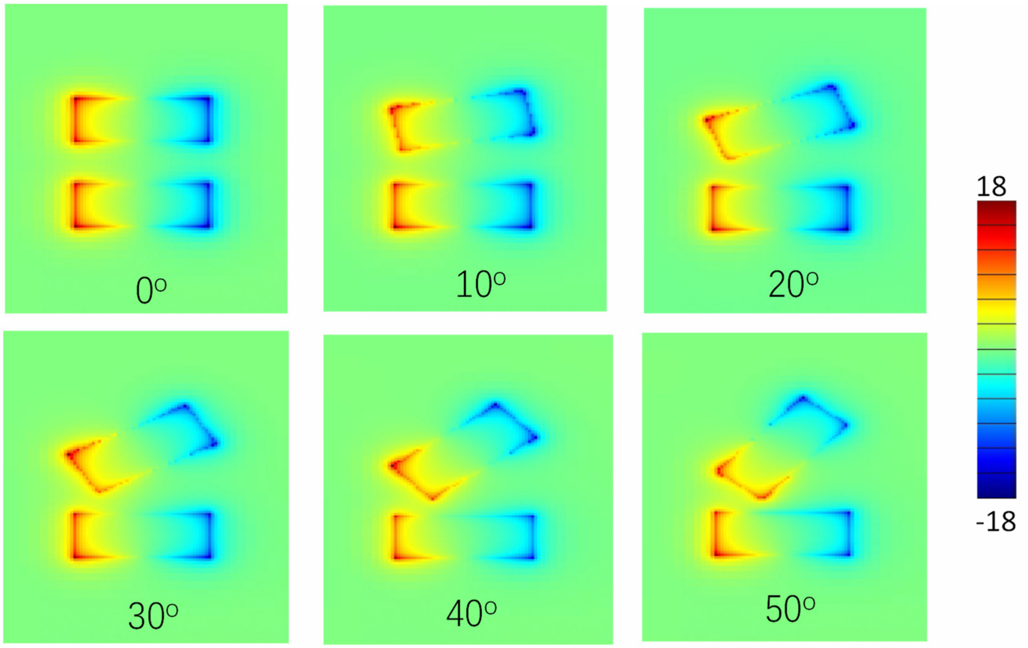


Figure S1. The simulated Re(Ez) patterns of the plasmonic mode (1100nm) with the nanorod rotation angle of 0, 20 and 50 degrees.

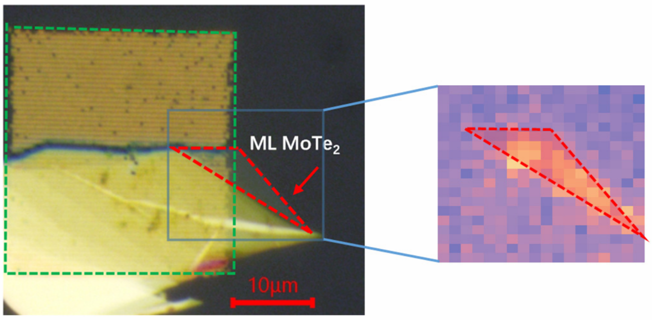


Figure S2. Image of the device and the corresponding PL mapping at the wavelength of 1140nm.

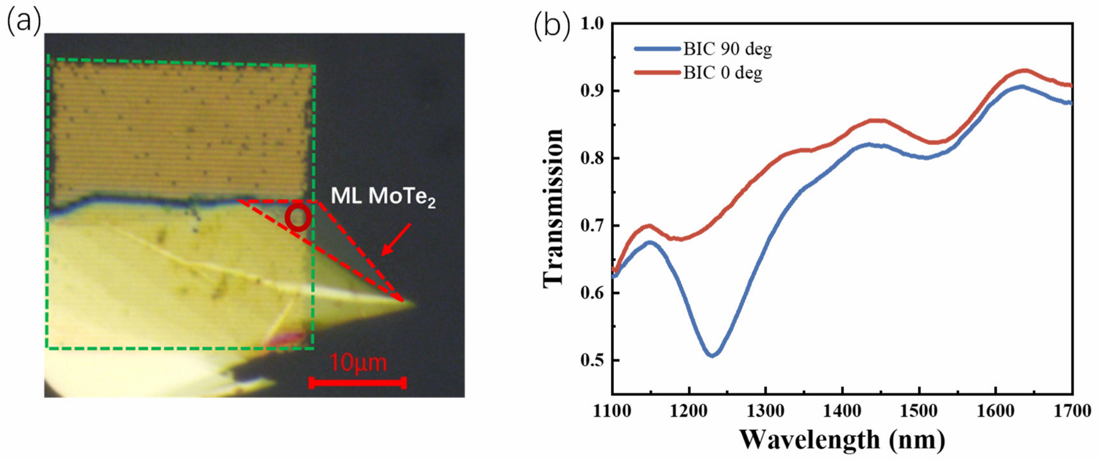


Figure S3. (a) Image of the device.For transmission measurement, the light spot is incident on the red circle, which is region of ML $MoTe_2$ covering by plasmonic BIC structure. (b) the transmission spectrum of the ML $MoTe_2$ covering by plasmonic BIC structure.

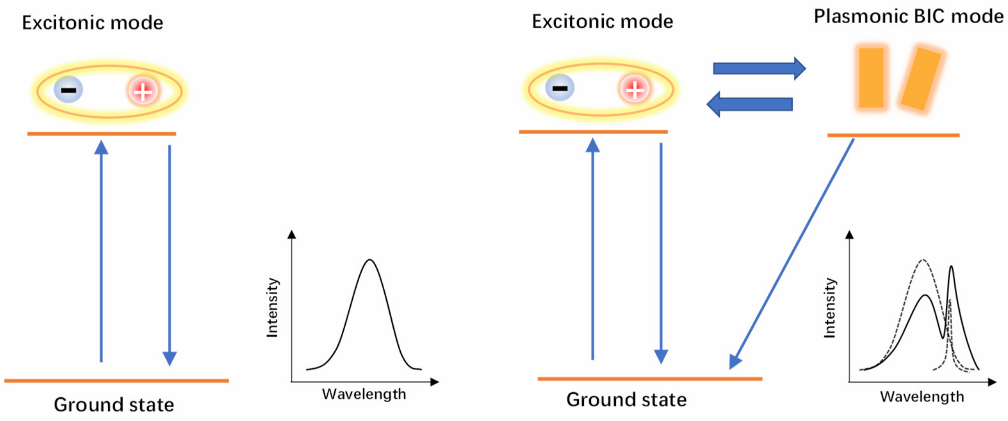


Figure S4. The diagram for PL spectrum of $MoTe_2$ without and with plasmonic BIC mode. Without coupling, the PL peak reflects the excitonic transition. When the coupling with plasmonic BIC mode, exciton will transfer energy to plasmonic mode, which open the plasmonic BIC transition channel. These two transitions will interfere with each other and lead to the Fano-like line shape.
